# Vom *Schwachsinn* zur *Intelligenzminderung*: Einige Anmerkungen aus forensisch-psychiatrischer Sicht zur Ersetzung des juristischen Begriffs im Schuldfähigkeitsparagrafen 20 StGB

**DOI:** 10.1007/s00115-026-01987-y

**Published:** 2026-06-16

**Authors:** Dieter Seifert

**Affiliations:** Rechtswissenschaftliche Fakultät, Institut für Kriminalwissenschaften – Abteilung IV, Universität Münster, Bispinghof 24/25, 48143 Münster, Deutschland

## Hintergrund

Sprache dient der Verständigung. Treffen unterschiedliche Fakultäten aufeinander, gelingt dies erfahrungsgemäß nicht in jedem Fall. Stehen z. B. Jurist*innen und Psychiater*innen im fachlichen Austausch, kann es zu sprachlichen Ungenauigkeiten mit ggf. weitreichenden Folgen kommen. Erschwerend kommt hinzu, dass die Begrifflichkeiten der Internationalen statistischen Klassifikation der Krankheiten und verwandten Gesundheitsprobleme (ICD) der Weltgesundheitsorganisation (WHO) im Laufe der Zeit vielfältige Änderungen erfahren haben. Zudem wird von Auflage zu Auflage neu verhandelt, was als normal und was als psychisch krank bzw. gestört definiert wird. Folglich muss auch die (terminologische) Entwicklung im beruflichen Miteinander erklärt werden.

Sprache vermittelt überdies Haltung und Einstellung zu einer Sache oder zu seinem Gegenüber und prägt unser Denken. Daher ist es legitim, sich mit möglichen Sprachverwirrungen bei der interdisziplinären Zusammenarbeit von Strafjustiz und forensischer Psychiatrie etwas genauer zu befassen. Aufgabe der Psychiatrie ist, bei den Bezeichnungen diagnostischer Kategorien und den Beschreibungen des Inhaltes der Kategorien eine wohl überlegte und behutsame Wortwahl zu treffen, ggf. Begriffe und Formulierungen zu überdenken und der sonstigen Sprachentwicklung zeitnah anzupassen. Wenngleich sich die Deutsche Gesellschaft für Psychiatrie und Psychotherapie, Psychosomatik und Nervenheilkunde (DGPPN) als größte Fachgesellschaft bereits seit Jahren gegen die Diskriminierung psychisch Kranker engagiert, sind Vorbehalte nach wie vor unübersehbar. So möchten selbst heutzutage 30 % der Bevölkerung einen Menschen mit Schizophrenie weder als Nachbarn noch als Arbeitskolleg*innen haben [18].

Eine Erwartung an den Gesetzgeber sollte sein, die Erkenntnisse und Erfahrungen der Psychiatrie – auch auf der terminologischen Ebene – in den Gesetzestexten zu berücksichtigen, was jahrzehntelang allenfalls bedingt der Fall war. So hatten sich im Schuldfähigkeitsparagrafen (§ 20 Strafgesetzbuch [StGB]) trotz mehrerer Novellierungen des Strafrechts die Begriffe „Schwachsinn“ und „seelische Abartigkeit“ bis zum 31.12.2020 gehalten. Die Frage darf erlaubt sein, wieso diese nicht bereits früher geändert wurden, zumal sich in dem langen Zeitraum die Sprache weiterentwickelt hat und beide Begriffe unverkennbar einen stigmatisierenden Charakter besitzen.

## Diagnostische Beschreibungen von Menschen mit Intelligenzminderungen

In keinem anderen Bereich der Medizin hat man sich mit primär fachlich formulierten Diagnosen derart schwergetan wie in der Psychiatrie. So sprach man im alten römischen Recht von „furiosi“ („die Wütenden“), „mente capti“ („die Verblödeten“) und „dementes“ („die Toren“). Für Menschen mit intellektuellen Beeinträchtigungen hören sich manche diagnostischen Kategorien und deren Beschreibungen bis in die nahe Vergangenheit als zumindest bedenklich an. Noch in der deutschsprachigen Ausgabe der ICD‑9 (Bundesministerium für Jugend, Familie, Frauen und Gesundheit 1986) wurden diese Störungsbilder unter der Überschrift „Oligophrenien“ (Nr. 317 bis 319) geführt und in die verschiedenen Schweregrade von *Schwachsinn* differenziert [4].

Der aus dem Griechischen stammende Terminus *Oligophrenie* findet sich bereits in dem über 100 Jahre alten Psychiatrielehrbuch von Eugen Bleuler [2] und bedeutet wörtlich übersetzt „wenig Seele“. Zum damaligen Zeitpunkt hieß die Schizophrenie noch „Dementia praecox“, die Demenz wurde unter der Überschrift „Altersblödsinn“ und hirnorganische Störungsbilder in dem Kapitel „Irresein bei Hirnerkrankungen“ abgehandelt. Zudem wurden Patient*innen mit solchen Persönlichkeitsstörungen (früher Psychopath*innen), die heute dem Cluster B zugeordnet würden, also gehäuft im Maßregelvollzug angetroffen werden, als „Gesellschaftsfeinde“ oder „moralische Idioten“ tituliert. Im Laufe der folgenden Jahrzehnte lässt sich bei nahezu allen psychiatrischen Störungsbildern eine Versachlichung bzw. Anpassung der diagnostischen Begriffe an die gebräuchliche Sprache erkennen. Wenngleich nicht verkannt wird, dass mit der Zeit einige wissenschaftliche Begriffe der Psychiatrie Eingang in die Umgangssprache fanden, bleibt ebenso festzustellen, dass insbesondere bei dieser Diagnosegruppe die sprachliche Anpassung vergleichsweise spät erfolgte. Möglicherweise wird diese verzögerte sprachliche Angleichung auch damit zu tun haben, dass sich die Psychiatrie mit dieser Patient*innengruppe lange Zeit weniger intensiv, fast nachlässig beschäftigt und das Feld weitgehend der Pädagogik überlassen hat.

Erst die deutschsprachige Ausgabe der ICD-10 übersetzte die Bezeichnung „Mental Retardation“ für die Kategorie F7 mit „Intelligenzminderung“ [5]. Die Kategorie gliederte sich in Schweregrade (leichte, mittelgradige, schwere und schwerste). Die deutschsprachige Ausgabe der 2019 verabschiedeten ICD-11 ersetzt nunmehr den Begriff „Intelligenzminderung“ durch den Begriff „Störungen der Intelligenzentwicklung“. Die jeweiligen Schweregrade sind gegenüber der ICD-10 nur unwesentlich sprachlich modifiziert worden [3]. Auch das DSM‑5 (Diagnostic and Statistical Manual of Mental Disorders 5) verwendet seit 2013 den Begriff „Intellectual Disability (Intellectual Developmental Disorder)“ und löst damit die frühere Bezeichnung „Mental Retardation“ ab [1]. Dort wird die Schweregradeinteilung nicht primär nach IQ-Werten durchgeführt, stattdessen werden die einzelnen Fertigkeiten im sozialen sowie Alltagsbereich detailliert beschrieben.

Die jeweiligen Novellierungen liefern somit einen Beitrag zur Entstigmatisierung dieser Diagnosegruppen und können zudem als Gegenpol zu der lange Zeit vorherrschenden Annahme des therapeutischen Nihilismus verstanden werden. Im Übrigen weisen die vielen begrifflichen Umbenennungen dieser Störungen auf die Komplexität und Schwierigkeiten hin, einen wissenschaftlich korrekten und gleichzeitig wertfreien Begriff zu finden.

Mit der Umformulierung des Schuldfähigkeitsparagrafen (§ 20 StGB) u. a. im Hinblick auf die Ersetzung des Begriffs Schwachsinn durch den Begriff Intelligenzminderung durch das Sechzigste Gesetz zur Änderung des Strafgesetzbuches vom 30.11.2020 ist nunmehr eine Angleichung an die Terminologie der ICD-10 erreicht, die den Dialog zwischen Strafjustiz und Psychiatrie zweifelsohne erleichtert hat. Allerdings scheinen einige Jurist*innen unverändert an überholten Begriffen festhalten zu wollen. So ist in der 71. Auflage des Beck’schen Kurzkommentars zum StGB, ein in der Strafrechtspraxis geltendes Standardwerk [7], das dritte Eingangsmerkmal des § 20 StGB zwar in Intelligenzminderung abgeändert; im Weiteren ist aber immer noch beispielsweise von persönlichkeitsgestörten debilen Personen (§ 20 StGB Rn. 35a) die Rede.

Dass die sprachliche Modernisierung bei Patient*innen mit Störungen der Intelligenzentwicklung ebenso in der psychiatrischen Terminologie – anders als bei den anderen großen Störungsbildern – nur zögerlich erfolgt ist, könnte auch darin begründet sein, dass in diesem Bereich gleichfalls das wissenschaftliche Engagement hinterherhinkt [15]. Dies gilt insbesondere für den Bereich der Therapieforschung. Hier bleibt zwischen der allgemeinpsychiatrischen Versorgung einschließlich der Behindertenhilfe und dem forensischen Kontext zu differenzieren. Während im erstgenannten Bereich bereits seit Jahren eine Reihe an Fachliteratur existiert (u. a. [10, 12]), sind nur vereinzelte wissenschaftliche Beiträge zur Begutachtung dieser forensischen Patientengruppe erschienen [8]; an empirischen Studien existiert bislang nur eine [16].

Zum besseren Verständnis sollen hier einige wenige charakteristische Eckpunkte dieser forensischen Diagnosegruppe aufgeführt werden:

- Der Anteil im Maßregelvollzug (§ 63 StGB) beträgt zwischen 5 und 12 %.
- Die mittlere Unterbringungsdauer ist im Vergleich zu anderen Diagnosegruppen auch heute noch länger, wenngleich die Straftatschwere tendenziell geringer ist; dieser Unterschied war Mitte der 1980er-Jahre noch wesentlich größer [9]: Damals waren sie im Mittel fast doppelt so lange untergebracht (11,5 vs. 6,3 Jahre).
- Die juristische Beurteilung der Schuldfähigkeit hat sich im Laufe der letzten vier Jahrzehnte signifikant verändert: Während in den 1980er-Jahren der Anteil der Schuldunfähigen bei 91,7 % lag, betrug er ein Jahrzehnt später 72 % und derzeit nur noch 36,3 % [16].
- Darüber hinaus konnte gezeigt werden, dass bei den Gutachter*innen z. T. erhebliche Unsicherheiten hinsichtlich diagnostischer Zuordnung und erst recht der forensischen Beurteilung von Steuerungs- und Einsichtsfähigkeit sowie der Legalprognose bestehen. Diese Unsicherheiten spiegeln sich desgleichen in den Urteilstexten der untersuchten Stichprobe wider [16], was vereinzelt zu erstaunlichen Fehleinschätzungen führte [14].

Ebenso haben strukturierte Überlegungen zur Therapie dieser Patient*innengruppe Seltenheitswert [11]. Auch mangelt es unverändert an forensischen Kliniken, die sich auf diese Patient*innen spezialisiert haben [13]. Eine solche Differenzierung scheint schon deswegen notwendig, um ein auf das jeweilige Störungsbild ausgerichtetes Therapieangebot herauszuarbeiten, mit dem langfristigen Ziel, die überlangen Unterbringungsdauern der Patient*innengruppe zu reduzieren. Gesetzesnovellierungen allein reichen erfahrungsgemäß nicht aus.

## Fazit

Nach Jahrzehnten der sprachlichen Divergenz beim dritten Eingangsmerkmal des § 20 StGB, die in der forensischen Praxis bzw. im Gerichtssaal mutmaßlich zu einigen Missverständnissen bis hin zu Fehlurteilen geführt haben dürfte, ist es vor fünf Jahren mit der Novellierung des Strafgesetzbuches gelungen, endlich eine begriffliche Anpassung des Gesetzestextes an die psychiatrische Terminologie zu erreichen. Im Übrigen ist diese Begriffsänderung nicht zuletzt aus Sicht der Betroffenen und ebenso der Angehörigen längst überfällig gewesen. Die vollzogene Änderung der Terminologie in der ICD-11, die aus klinisch-psychiatrischer und entwicklungspsychologischer Sicht durchaus sinnvoll ist, führt jedoch erneut zu einer terminologischen Diskrepanz zwischen Psychiatrie und Gesetzestext. Anders ausgedrückt: Die Einführung der fachlich zeitgemäßeren Terminologie Ende 2020 erfolgte zu einem Zeitpunkt, als die WHO mit der Verabschiedung der ICD-11 im Jahre 2019 schon den neuen Begriff implementiert hatte [17]. Kritisch anzumerken ist zudem, dass man – als sinnvolle Konsequenz – im Rahmen dieser Gesetzesnovellierung ergänzend hätte darüber nachdenken können, ob die jahrzehntelange juristische Differenzierung in Intelligenzminderungen, die auf eine organische Ursache zurückzuführen sind (erstes Eingangsmerkmal des § 20 StGB – „krankhafte seelische Störung“), und solche, bei denen eine angeborene, also ohne nachweisbare Ursache bestehende Intelligenzminderung (drittes Eingangsmerkmal) vorliegt, noch zeitgemäß und wissenschaftlich begründbar ist. In Anbetracht der in den letzten Jahren zu beobachtenden neurobiologischen bzw. -radiologischen und molekulargenetischen Fortschritte ist zu erwarten, dass demnächst bei konsequenter Anwendung dieser Differenzierung immer seltener intelligenzgeminderte Rechtsbrecher*innen dem dritten Eingangsmerkmal zugeordnet werden dürften.

Um in Zukunft sprachliche Ungereimtheiten mit eventuell folgenschweren juristischen (und therapeutischen) Konsequenzen zu verhindern, sollte nunmehr vorgebeugt werden. Dies ist als Plädoyer für eine engere Zusammenarbeit zwischen Strafjustiz und Forensik und ebenso der Allgemeinpsychiatrie [6] zu verstehen: Anzustreben bzw. zu erweitern sind gemeinsame Fortbildungen z. B. bei der Deutschen Richterakademie und den Landesjustizakademien sowie den jeweiligen Ärztekammern. Darüber hinaus empfiehlt sich, bereits im Studium den interdisziplinären fachlichen Dialog zu etablieren, wie das bereits in einigen juristischen Fakultäten mit dem Schwerpunkt „Kriminalwissenschaften“ der Fall ist.

## Data Availability

Alle dieser Arbeit zugrunde liegenden Daten sind in diesem Artikel enthalten.
